# Scalable Culturing of Primary Human Glioblastoma Tumor-Initiating Cells with a Cell-Friendly Culture System

**DOI:** 10.1038/s41598-018-21927-4

**Published:** 2018-02-23

**Authors:** Qiang Li, Haishuang Lin, Jack Rauch, Loic P. Deleyrolle, Brent A. Reynolds, Hendrik J. Viljoen, Chi Zhang, Chi Zhang, Linxia Gu, Erika Van Wyk, Yuguo Lei

**Affiliations:** 1 Department of Chemical and Biomolecular Engineering, University of Nebraska, Lincoln, Nebraska USA; 2 Biomedical Engineering Program, University of Nebraska, Lincoln, Nebraska USA; 3 Department of Neurosurgery, University of Florida College of Medicine, McKnight Brain Institute, Gainesville, Florida USA; 4 School of Biological Science, University of Nebraska, Lincoln, Nebraska USA; 5 Department of Radiation Oncology, College of Medicine, University of Nebraska Medical Center, Omaha, Nebraska USA; 6 Department of Mechanical & Materials Engineering, University of Nebraska, Lincoln, Nebraska USA; 7 CellGro Technologies, LLC, Adams, NE USA; 8 Mary and Dick Holland Regenerative Medicine Program, University of Nebraska Medical Center, Omaha, Nebraska USA; 9 Fred & Pamela Buffett Cancer Center, University of Nebraska Medical Center, Omaha, Nebraska USA

## Abstract

Glioblastoma is the most aggressive and deadly brain cancer. There is growing interest to develop drugs that specifically target to glioblastoma tumor-initiating cells (TICs). However, the cost-effective production of large numbers of high quality glioblastoma TICs for drug discovery with current cell culturing technologies remains very challenging. Here, we report a new method that cultures glioblastoma TICs in microscale alginate hydrogel tubes (or AlgTubes). The AlgTubes allowed long-term culturing (~50 days, 10 passages) of glioblastoma TICs with high growth rate (~700-fold expansion/14 days), high cell viability and high volumetric yield (~3.0 × 10^8^ cells/mL) without losing the stem cell properties, all offered large advancements over current culturing methods. This method can be applied for the scalable production of glioblastoma TICs at affordable cost for drug discovery.

## Introduction

Glioblastoma is the most aggressive and deadly adult brain cancer^1–3^. The standard treatments including surgery, chemotherapy and radiotherapy cannot cure the disease. The median survival time is about 15 months^4^. Studies attribute the high drug resistance and recurrence of glioblastoma to the existence of a small population of cells termed the tumor-initiating cells (TICs) within the tumor mass^5–7^. TICs have the stem cell properties including self-renewal and differentiation. They also have high resistance to the standard treatments^8^. The survived TICs expand and differentiate to re-initiate tumors, resulting in recurrence. Therefore, researchers have suggested that killing or differentiating these glioblastoma TICs represents a promising approach to treat or cure glioblastoma^9^.

Human glioblastoma TICs have been successfully isolated through neurosphere culturing or using surface markers such as CD133^1,10^, CD15^11^ and ABCG2^12,13^, etc. Some recent studies showed that using these markers, such as CD133, to define glioblastoma TICs is still controversial^11,14–16^. The glioblastoma TICs also express Nestin, Sox2, CD44, or Olig2^2,17^. These cells can be cultured for a long time and differentiated into astrocytes, neurons and oligodendrocytes *in vitro*^1,2,7^. When transplanted into immune deficiency mice, they can form tumors that are similar to the primary tumors found in the patients^7,18^. These TICs are very valuable for developing new drugs for treating glioblastoma. For instance, many studies have already applied these cultured TICs for drug discovery and development^19–23^. Drug discovery requires large numbers of cells. For example, it is estimated that ~1 × 10^10^ cells are needed to screen a one-million-compound library once^20^. Advances in combinatorial chemistry, noncoding RNAs and investigations of complex signaling and transcriptional networks have given rise to large libraries that can be screened^20^. Massive numbers of TICs are, therefore, needed to deliver on the biomedical promise of these cells.

However, culturing high quantity and high quality glioblastoma TICs with current cell culturing methods including the two dimensional (2D) adherent culturing and three dimensional (3D) suspension culturing remains very challenging. Pollard *et al*. reported that glioblastoma TICs could be cultured on 2D surface for long-term with good cell viability, proliferation and maintenance of stem cell phenotypes^2^. A number of reports have shown that glioblastoma TICs could be cultured as aggregates (or neurospheres) with good maintenance of cell phenotype^17,24–26^. Although these culture systems work well for preparing cells for basic science research, they are considered not suitable for producing cells at large scales^27^. 2D culturing is labor-, space- and reagent-consuming^28–30^. Additionally, a 2D environment is very different from an *in vivo* environment. It can induce significant DNA instability and positively select cells gaining survival and growth privileges due to the genetic aberrations^31–34^. The neurosphere method usually cultures TICs at low density (e.g. <1 × 10^6^ cells/mL)^18^, requiring large culture volume to generate cells at large-scale.

**Figure 1:**
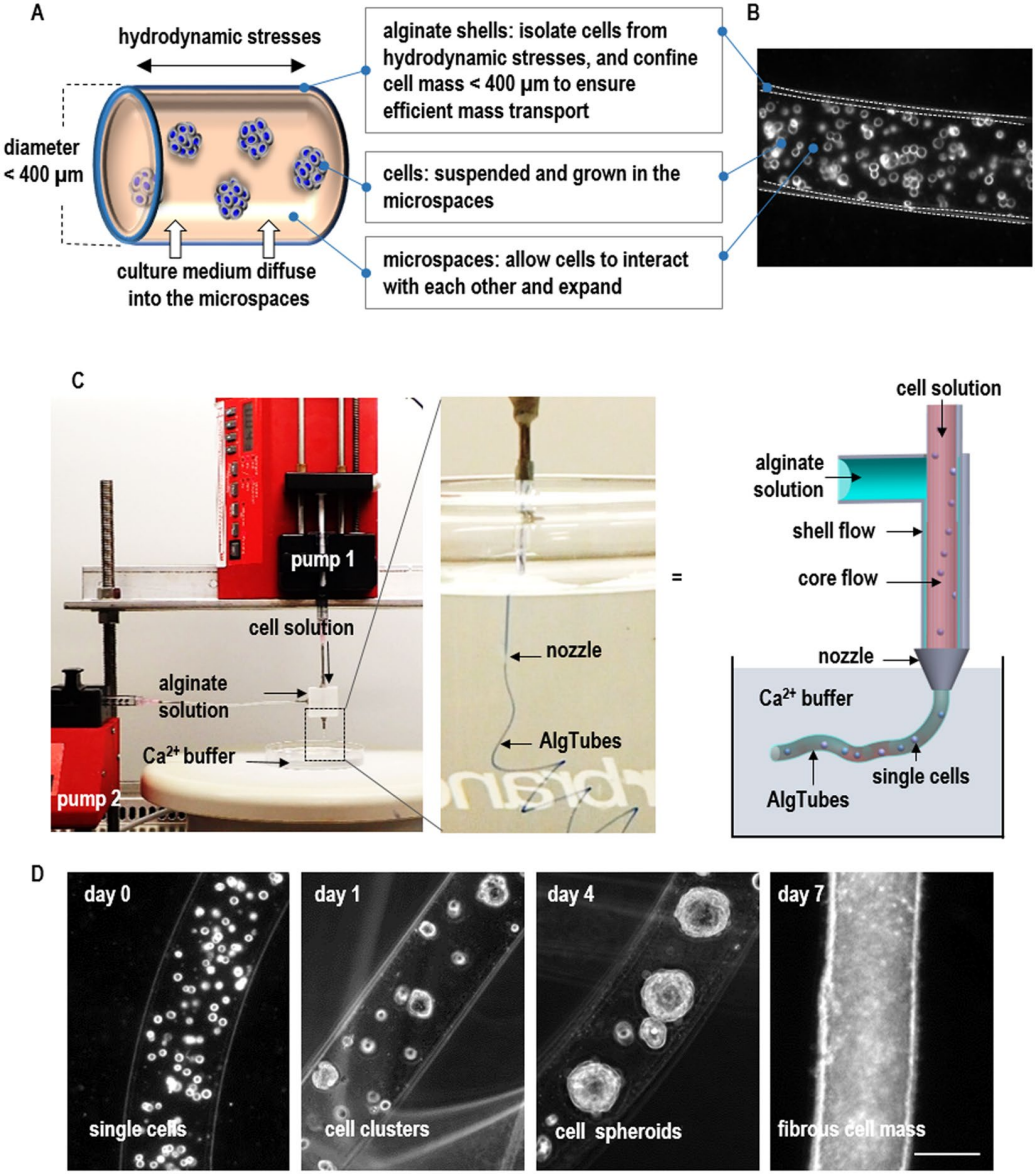
Culturing glioblastoma tumor-Initiating cells (TICs) in alginate hydrogel tubes (AlgTubes). (**A**,**B**) Glioblastoma TICs were suspended and cultured in microscale alginate hydrogel tubes that were suspended in the cell culture medium in a culture vessel. The tubes protected cells from hydrodynamic stresses in the culture vessel and confined the cell mass less than 400 µm (in radial diameter) to ensure efficient mass transport. They also provided microspaces for cells to interact with each other and expand. Cell culture medium could efficiently diffuse through the alginate hydrogel shell. An illustration (**A**) and microscope picture (**B**) of an AlgTube. (**C**) To process AlgTubes, a cell and an alginate solution was pumped into the central channel and side channel of a micro-extruder, respectively, to form coaxial core-shell flows that were extruded through the nozzle of the micro-extruder into a CaCl_2_ buffer. The shell alginate flow was instantly crosslinked by the Ca^2+^ ions to form an alginate hydrogel tube. (**D**) In AlgTubes, individual cells first associated to form small cell clusters. Subsequently, cells proliferated and the small cell clusters expanded to form fibrous cell mass. Scale bar: 200 µm.

We here report a novel and scalable cell culture system to address this challenge. With this technology, TICs are suspended and cultured in microscale alginate hydrogel tubes (or AlgTubes) that are suspended in the cell culture medium in a culture vessel (Fig. 1A,B). We showed that, under optimized culture conditions, TICs from multiple patients could be cultured with high cell viability, growth rate (~700-fold expansion/14 days) and volumetric yield (~3.0 × 10^8^ cells/mL), all offered large advancements over the current culturing methods. Alginate hydrogels are used for making this culture system because^35^ they: (1) can be quickly processed in large scales with the extruder; (2) can be easily dissolved to release the product; (3) allow quick nutrient diffusion through the hydrogel shell; (4) are mechanically and chemically stable for cell cultures; and (5) are transparent, allowing optical monitoring. Additionally, alginates are affordable and available in large quantities. They have no toxicity^36^. This technology can be applied for the mass production of glioblastoma TICs at affordable cost for drug discovery.

## Results

### The AlgTubes cell culture system

*In vivo*, glioblastoma TICs reside in 3D microenvironments that have plenty of cell-cell and cell-ECM (extracellular matrix) interactions, sufficient supply of nutrients, oxygen and growth factors and no or minimal hydrodynamic stresses^37^. We designed the AlgTubes to mimic these *in vivo* microenvironments for culturing glioblastoma TICs. The hydrogel tubes created cell-friendly microspaces that allowed TICs to interact with each other and expand. Meanwhile, the tubes protected TICs from hydrodynamic stresses in the culture vessel and confined the cell mass less than 400 µm (in radial diameter) to ensure efficient mass transport during the entire culture (Fig. 1A,B).

A custom-made micro-extruder was made to process glioblastoma AlgTubes (Fig. 1C). A 2% hyaluronic acid (HA) solution with single dissociated glioblastoma TICs was pumped into the central channel and a 1.5% alginate solution was pumped into the side channel of the extruder, respectively (Fig. 1C). The two solutions as coaxial core-shell flows were extruded into a 100 mM Ca^2+^ buffer, which instantly crosslinked the alginate flow to form hydrogel shells to make AlgTubes. In the AlgTubes, individual TICs associated with neighboring cells to form small cell clusters within 24 hours. Subsequently, these clusters grew and eventually merged to form fibrous cell masses (Fig. 1D). To passage cells, AlgTubes were dissolved with 0.5 mM ethylenediaminetetraacetic acid (EDTA) solution (5 mins at room temperature). The released cell masses were treated with 0.05% Trypsin for 10 minutes at 37 °C and dissociated into single cells for the following passage or analysis.

### Culturing primary glioblastoma TICs in AlgTubes

To evaluate the general applicability, three patient-derived primary glioblastoma TICs (L0, L1 and L2) were cultured in the AlgTubes. These cells were established as described in our previous publication^7^. They expressed neural stem cell markers including Nestin, SOX2 and Olig2, while very few of them expressed the differentiation markers such as Tuj1 and GFAP (Fig. S1). They also expressed TIC markers mentioned in the literature such as CD133, CD15 and CD44. About 4.6%, 18.5% and 99.8% of L0 cells were CD133+, CD15+ and CD44+, respectively. About 14.1%, 18.4% and 99.7% of L1 cells were CD133+, CD15+ and CD44+, respectively. And about 3.9%, 16.6% and 99.8% of L2 cells were CD133+, CD15+ and CD44+, respectively (Fig. S1). The results agree well with our previous publication^7^.

**Figure 2:**
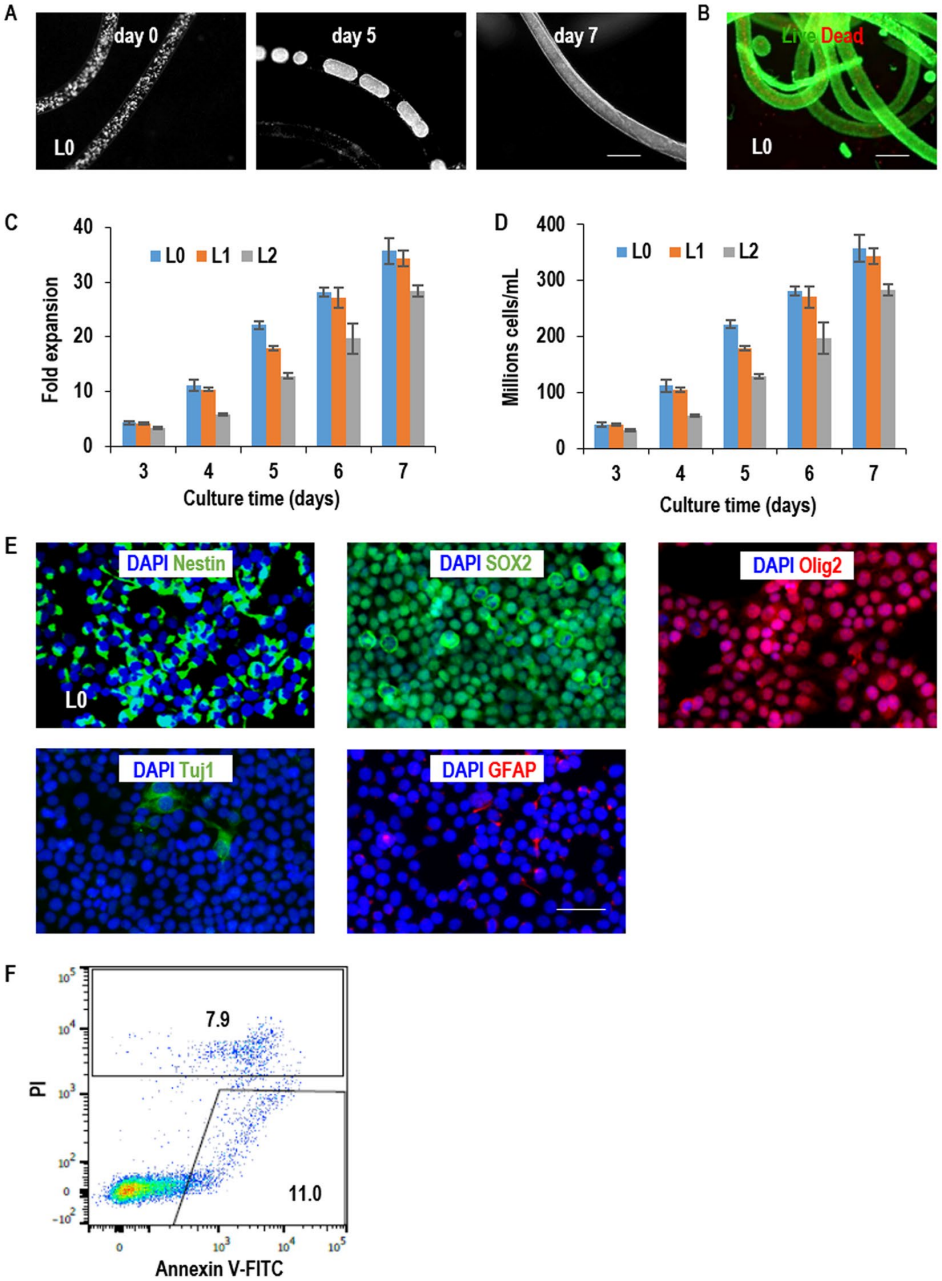
Culturing glioblastoma TICs in AlgTubes at passage 1. (**A**) Microscopy images of glioblastoma TICs (L0) in AlgTubes on day 0, 5 and 7. (B) Live/dead staining of day 7 cells in AlgTubes. (**C**,**D**) The expansion fold and volumetric yield of TICs from day 3 to day 7. When seeded at 1 × 10^7^ cells/mL, L0, L1 and L2 expanded 36-, 34- and 28-fold, yielding 3.6 × 10^8^, 3.4 × 10^8^ and 2.8 × 10^8^ cells/mL by day 7, respectively. (**E**) Immunostaining of the cultured TICs. Cells were released from AlgTubes on day 7 and plated on a Laminin-coated plate overnight before fixing and staining. Majority of L0 cells were Nestin+, SOX2+ and Olig2+. Few L0 cells were Tuj1+ and GFAP+. (**F**) Cell death was evaluated by Annexin V and PI staining after cultured in AlgTubes for 7 days. Error bars represent the standard deviation (n = 3). Scale bar: (**A**,**B**) 400 µm, (**E**) 50 µm.

In AlgTubes, TICs expanded and formed fibrous cell masses with very few dead cells (Figs 2A,B, S2A,B). When seeded at 1 × 10^7^ cells/mL, L0, L1 and L2 expanded ~36-, 34- and 28-fold, yielding 3.6 × 10^8^, 3.4 × 10^8^ and 2.8 × 10^8^ cells/mL, respectively, by day 7 (Fig. 2C,D). Cells could be seeded at lower densities. When seeded at 2 × 10^6^ cells/mL, L0, L1 and L2 cells expanded ~175-, 170- and 139-fold to yield 3.5 × 10^8^, 3.4 × 10^8^ and 2.8 × 10^8^ cells/mL by day 12 (Fig. S3). When seeded at 5 × 10^5^, L0, L1 and L2 cells expanded ~683-, 640- and 500-fold to yield 3.4 × 10^8^, 3.2 × 10^8^ and 2.5 × 10^8^ cells/mL by days 14 (Fig. S3). Majority of TICs expressed Nestin, SOX2 and Olig2, while very few of them expressed the differentiation markers Tuj1 and GFAP after the 7-day culturing (Figs 2E and S2C). Live/dead cell staining detected very few dead cells (Figs 2B and S2B) and this was confirmed with Annexin V and PI staining (Figs 2F and S2D). We did not see significant difference in terms of cell viability, cell growth rate, yield and TIC markers expression when TICs were cultured in AlgTubes with diameters in the range of 100 μm to 400 μm. In short, glioblastoma TICs from different patients could be efficiently expanded in the new culture technology.

### Long-term culturing of glioblastoma TICs in AlgTubes

**Figure 3:**
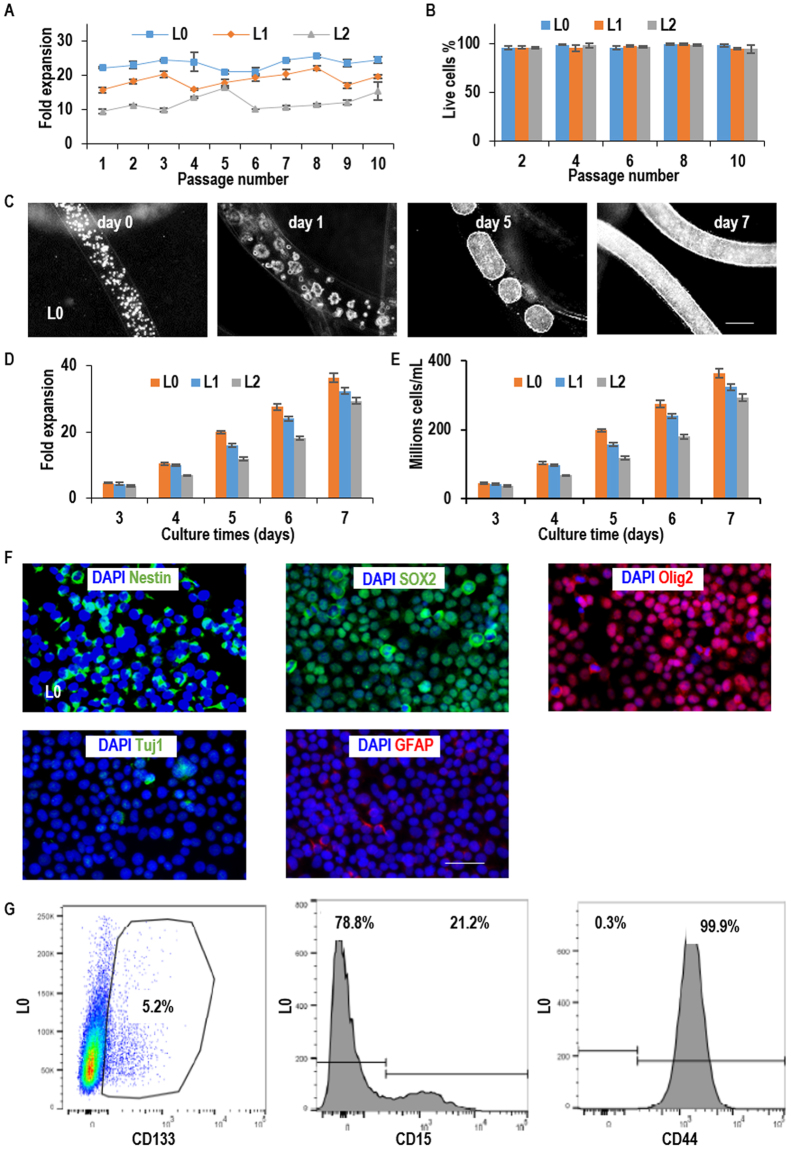
Long-term culturing of glioblastoma TICs in AlgTubes. (**A**) When seeded at 1.0 × 10^7^ cells/mL, L0, L1 and L2 expanded ~23-, 19- and 12-fold/passage/5 days, respectively, along a 10-passage culture with (**B**) cell viability was >95%. (**C**) Microscopy images of L0 cells on day 0, 1, 5 and 7 at passage 10. (**D**,**E**) The expansion fold and volumetric yield of TICs from day 3 to day 7 at passage 10. When seeded at 1 × 10^7^ cells/mL, L0, L1 and L2 expanded 36-, 32- and 29-fold, yielding 3.6 × 10^8^, 3.2 × 10^8^ and 2.9 × 10^8^ cells/mL by day 7, respectively. (**F**) Immunostaining of the cultured TICs. Cells were released from AlgTubes on day 7 at passage 10 and plated on a Laminin-coated plate overnight before fixing and staining. Majority of L0 were Nestin+, SOX2+ and Olig2+. Few L0 cells were Tuj1+ and GFAP+. (**G**) Flow cytometry analysis showed that 5.2% L0 cells were CD133+, 21.2% L0 cells were CD15+ and 99.9% L0 cells were CD44+. Error bars represent the standard deviation (n = 3). Scale bar: (**C**) 200 µm; (**F**) 50 µm.

To assess whether the AlgTubes support long-term culturing of glioblastoma TICs, all the three lines (L0, L1 and L2) were continuously cultured for 10 passages (e.g. about two months). Cells were passaged every 5 days. During the 10-passage culture, when seeded at 1.0 × 10^7^ cells/mL, L0, L1 and L2 expanded ~23-, 19- and 12-fold per passage per 5 days, respectively, with cell viability >95% (Fig. 3A,B). We re-evaluated the cell growth kinetics at passage 10 to study if the long-term culturing changed the cell growth rate or phenotype. When seeded at 1 × 10^7^ cells/mL, L0, L1 and L2 expanded ~36-, 32 and 29-fold, yielding 3.6 × 10^8^, 3.2 × 10^8^ and 2.9 × 10^8^ cells/mL, respectively, by day 7 (Fig. 3D,E). Majority of TICs expressed Nestin, SOX2 and Olig2, while very few of them expressed the differentiation markers Tuj1 and GFAP (Figs 3F and S4B). The percentage of CD133+, CD15+ and CD44+ cells at passage 10 were very similar to these of the starting materials (Figs 3G and S1,4C). Our qPCR data also showed there were no differences in Nestin, CD44, CD15 and CD133 mRNA expressions between passage 10 and passage 0 (Fig. S4D,E). These data indicate that there are no significant phenotypic changes during the long-term culturing.

**Figure 4:**
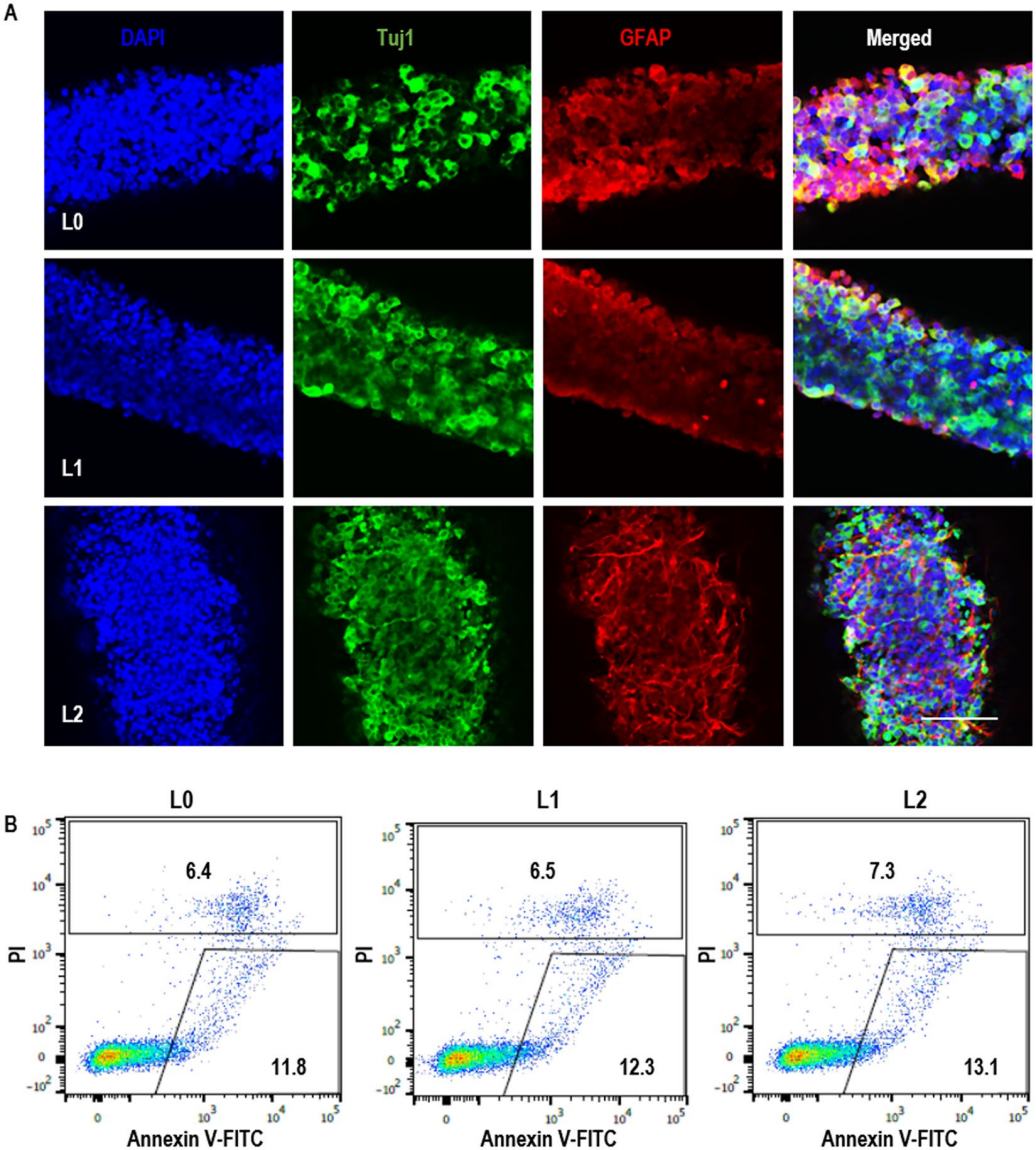
*In vitro* differentiation of glioblastoma TICs. (**A**) After 10 passages in AlgTubes, L0, L1 and L2 were spontaneously differentiated in AlgTubes for 2 weeks. Fibrous cell masses were fixed for staining. All TICs could be differentiated into Tuj1+ neurons and GFAP+ glial cells. **(B**) After 10 passages in AlgTubes, L0, L1 and L2 were cultured for 7 days after removing bFGF from the culture. Cell death was evaluated by Annexin V and PI staining for L0, L1 and L2. Scale bar: 100 µm.

**Figure 5:**
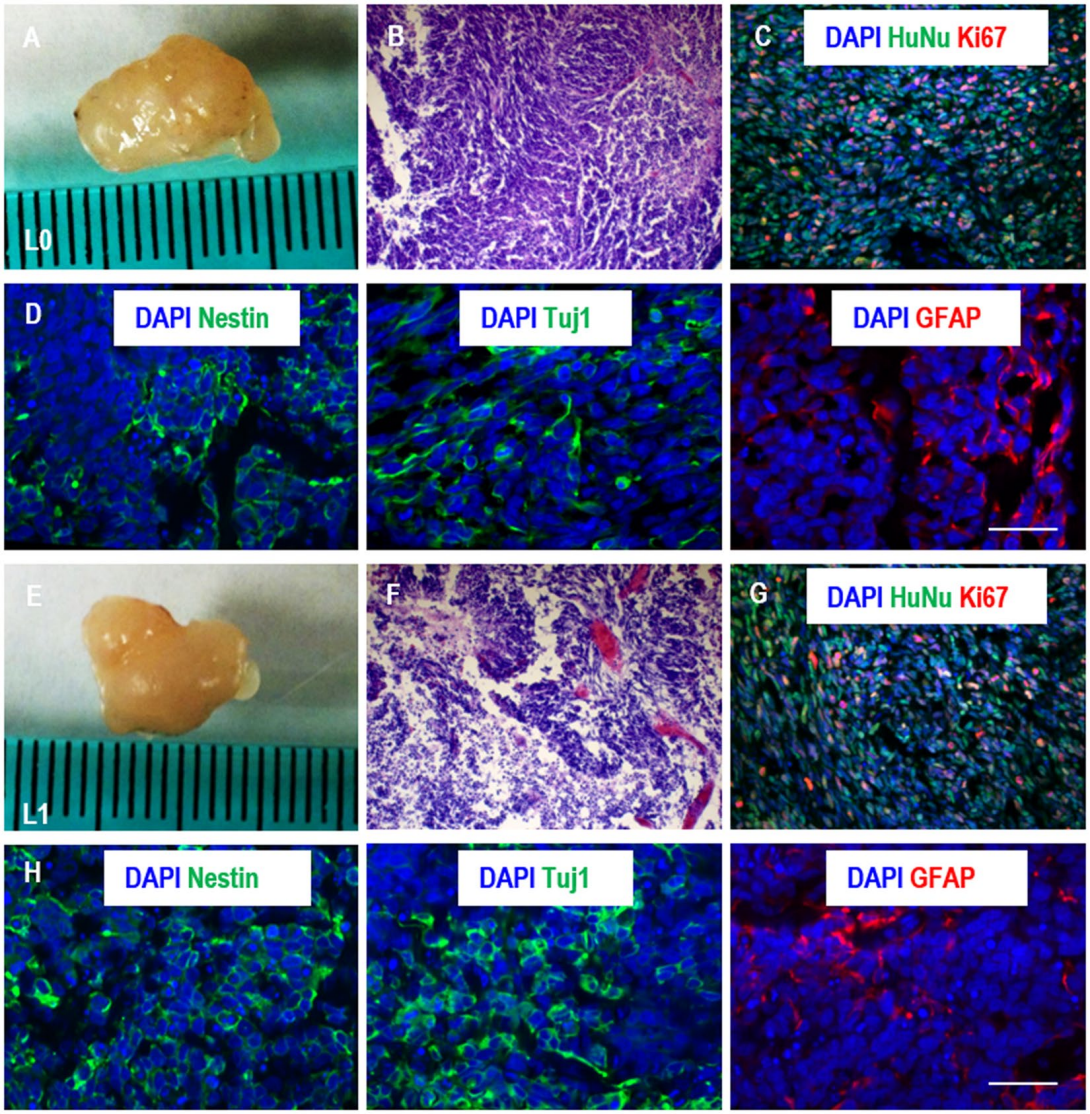
Xenotransplantation of glioblastoma TICs. After 10 passages in AlgTubes, TICs were transplanted subcutaneously to NOD-SCID mice. (**A**,**E**) Harvested tumors. (**B**,**F**) H&E staining of the tumor sections. (**C**,**G**) Large percentage of cells in the tumor tissue were human nuclear antigen (HuNu) positive human cells and were proliferating (Ki67+). (**D**,**H**) Nestin+ TICs, Tuj1+ neurons and GFAP+ glia cells were all found in the tumor tissue. Scale bar: 50 µm.

To confirm the stem cell identity of TICs after the long-term culturing, we withdrawn the bFGF factors from the culturing medium to initiate spontaneous differentiation of TICs within the tubes. After two weeks, all three TICs generated Tuj1+ neurons and GFAP+ glial cells (Figs 4A and S5). The removal of bFGFs did not induce significant cell death (Fig. 4B). These cells also successfully formed tumors when transplanted into immune-deficiency mice. Majority of the cells in the tumor were HuNu+ human cells and large percentages of cells were Ki67+ proliferating cells. Histological analysis showed Tuj1+ neurons, GFAP+ glial cells and Nestin+ TICs in all tumors (Fig. 5).

### Scalable culturing of glioblastoma TICs with AlgTubes in a bioreactor

**Figure 6:**
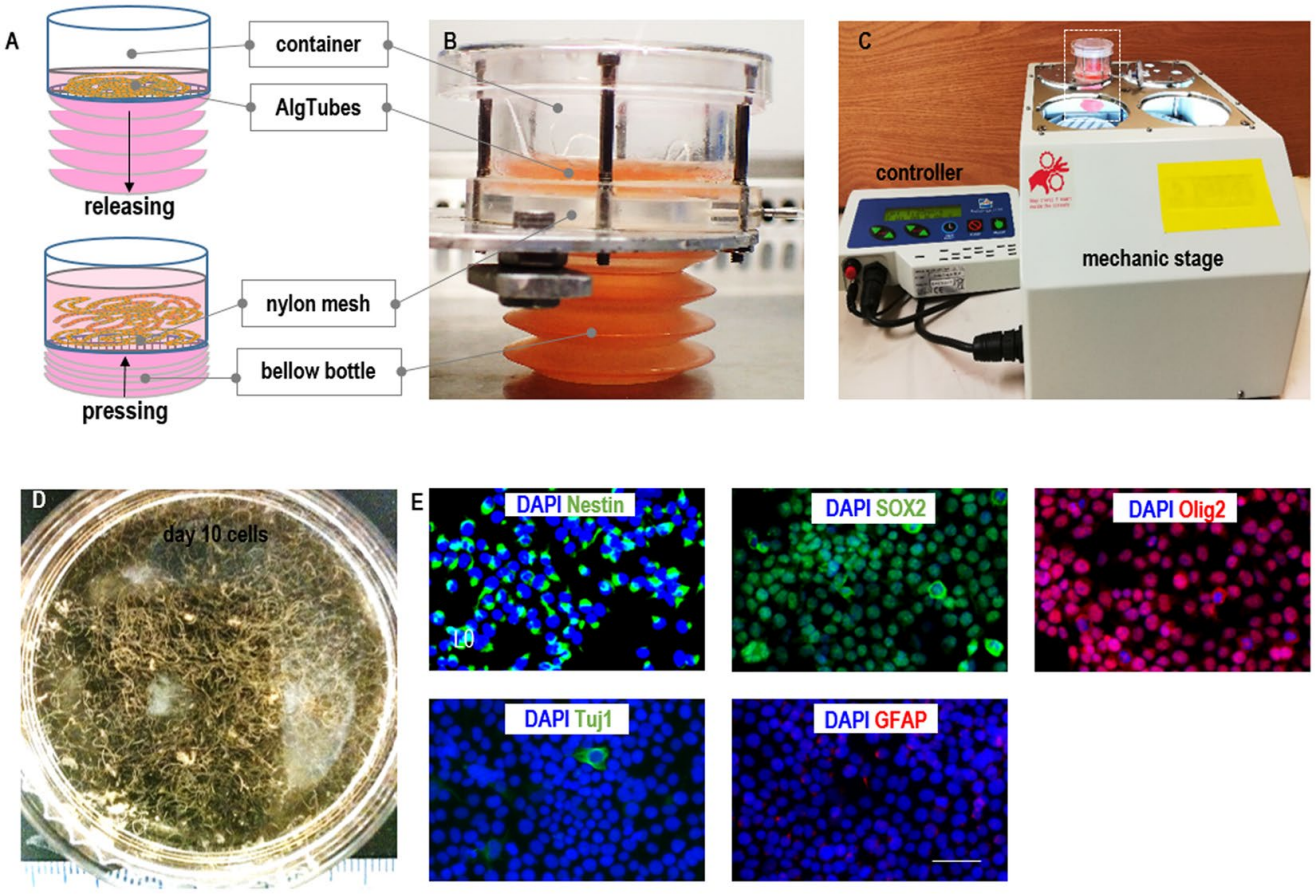
A prototype bioreactor for scalable TICs production. (**A**) The bioreactor contains a cylindrical container and a plastic bellow bottle, which are separated by a nylon mesh. AlgTubes with TICs are suspended in the cylindrical container and the medium is stored in the plastic bellow bottle that can be pressed to flow the medium into, or released to withdraw the medium from the container. (**B**,**C**) Images of the bioreactor. The mechanic stage is used to press and release the bellow bottle. The controller can be programmed for the pressing and releasing speed, as well as the duration of the interval between the pressing and releasing. (**D**) Glioblastoma TICs harvested from the bioreactor on day 10. TICs grew well and yielded ~3.0 × 10^8^ cells/mL. (**E**) Immunostaining of the cultured TICs. Majority of L0 were Nestin+, SOX2+ and Olig2+. Few L0 cells were Tuj1+ and GFAP+. Scale bar: 50 µm.

A prototype bioreactor was developed for the scalable manufacturing of glioblastoma TICs using AlgTubes (Fig. 6). The bioreactor contains a cylindrical container and a plastic bellow bottle, which were separated by a nylon mesh. AlgTubes with cells were suspended in the cylindrical container and the medium was stored in the plastic bellow bottle that could be pressed by a mechanic stage to flow the medium into, or released to withdraw the medium from the container (Fig. 6A,B). A mechanic stage was used to press and release the bellow bottle. A controller that can be programmed for the pressing and releasing speed, as well as the duration of the interval between the pressing and releasing was used to control the mechanic stage (Fig. 6C). Glioblastoma TICs grew well in the bioreactor and yielded ~3.0 × 10^8^ cells/mL (Fig. 6D). Majority of TICs expressed Nestin, SOX2 and Olig2, while very few of them expressed the differentiation markers Tuj1 and GFAP (Figs 6E and S6).

### Comparing culturing glioblastoma TICs in AlgTubes, 2D culturing, static 3D and dynamic 3D suspension culturing

**Figure 7:**
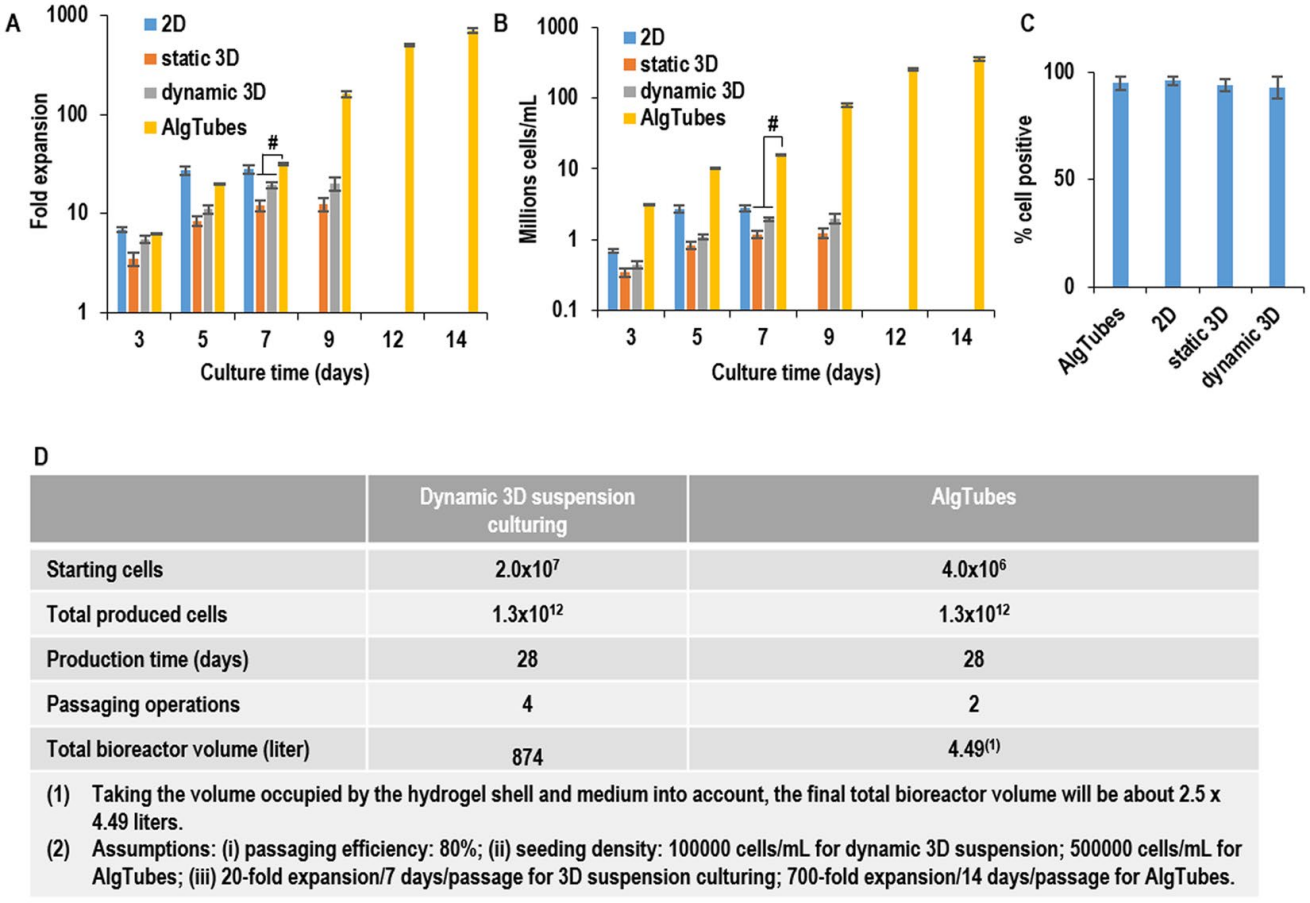
Comparing culturing glioblastoma TICs in AlgTubes, 2D culturing, static 3D and dynamic 3D suspension culturing. Glioblastoma TICs (L0) were seeded at 1 × 10^5^ cells/well (of 6-well plate) and cultured for 7 days in 2D culturing; seeded at 1 × 10^5^ cells/mL and cultured for 9 days in static 3D and dynamic 3D suspension; seeded at 5 × 10^5^ cells/mL and cultured for 14 days in AlgTubes. (**A**) The expansion fold, (**B**) volumetric yield and (**C**) % cell Nestin+ cells in the culture were quantified. (**D**) A comparative calculation of producing 1.3 × 10^12^ glioblastoma TICs. In a bioprocess using dynamic 3D suspension culturing (e.g. with stirred tank bioreactor), 1.3 × 10^12^ TICs are generated through series expansions of 2.0 × 10^7^ cell seeds with 4 passaging operations in 28 days. The total culture volume is ~874 liters. In a bioprocess using AlgTubes, 1.3 × 10^12^ TICs are generated from 4.0 × 10^6^ seeds with 2 passaging operation in 28 days. Total of 4.49 liters AlgTubes are needed. ^#^P < 0.05. Data are presented as mean ± SD of three replicates (n = 3). Note for 2D culturing, the unit for cell density in (**B**) is cells/well (of 6-well plate).

To show the superiority of AlgTubes for culturing glioblastoma TICs over current technologies, we directly compared expanding glioblastoma TICs (L0) in AlgTubes, 2D culturing, static 3D and dynamic 3D suspension culturing (Figs 7A–C and S7). For 2D culturing, 1 × 10^5^ cells/well were seeded in Laminin-coated 6-well plate. Cells reached confluent by day 5. L0 cells expanded ~27-fold to generate ~2.7 × 10^6^ cells per well of the 6-well plate. For static 3D suspension culturing, in which cells were suspended in culture medium at 1 × 10^5^ cells/mL without agitation, L0 cells formed aggregates with diameter in the range of 100 to 350 µm by day 7 and expanded ~12-fold to yield ~1.2 × 10^6^ cells/mL. In dynamic 3D suspension culture, in which cells were suspended in culture medium at 1 × 10^5^ cells/mL with agitation (~75 rotation per minute), L0 cells expanded ~20-fold to yield ~2 × 10^6^ cells/mL. In AlgTubes, L0 cells expanded ~710-fold to yield ~3.55 × 10^8^ cells/mL by 14 days when seeded at 5 × 10^5^ cells/mL. For all the cultures, majority of cells were Nestin+, SOX2+ and Olig2+. Few cells were Tuj1+ and GFAP+. In short, AlgTubes result in much higher expansion and volumetric yield than the current culture methods.

## Discussion

Our results showed TICs expanded around 25-fold, yielding ~2.5 × 10^6^ cells/well in 5 days in 2D culturing (Figs 7A,B and S7). In a typical drug screening, ~1 × 10^10^ glioblastoma TICs are needed to screen a library with 1 × 10^6^ compounds once. About seven hundreds of 6-well plates will be used to generate these cells. Maintaining these plates requires large incubator space, labor, time and cost. In addition, plate-to-plate and batch-to-batch variations are common in 2D culturing. Therefore, 2D culturing is generally considered not suitable for culturing large-scale cells^28–30^.

3D suspension culturing is considered promising for scaling up the cell production^28–30^. The neurosphere culture is commonly used for isolating and expanding glioblastoma TICs^17,24–26^. However, a significant problem with 3D suspension culturing is the uncontrolled cell agglomeration. Human cells usually have strong cell-cell interactions that make them aggregate^38,39^. Suspended cells tend to form large cell agglomerates (i.e., agglomeration), especially at high cell culture density. The agglomeration leads to inhomogeneity in cell aggregate size and is detrimental to cell culture^28^. For instance, the transport of nutrients, oxygen and growth factors to and the metabolic waste from, cells located at the center of large cell agglomerates (e.g., >400 µm diameter) become insufficient, leading to slow cell growth, apoptosis and phenotype changes^28,40^. Our results showed about 1 × 10^6^ TICs per milliliter of volume could be produced with 3D suspension culturing without agitation (Figs 7A,B and S7). With this yield, it will require about 10 liters culture volume to generate ~1 × 10^10^ glioblastoma TICs.

Agitation is generally applied to enhance the medium mixing and mass transport and reduce the cell agglomeration in 3D suspension culturing^41^. Our results showed TIC aggregates were much smaller and uniform under agitation (Fig. S7). However, agitation also generates complicated hydrodynamic conditions (e.g., the medium flow direction, velocity, shear force and chemical environment) that are spatially and temporally varied, resulting in locations with turbulence and critical stresses (e.g., near the vessel wall and impeller tip) that induce cell death and differentiation, leading to low cell viability, growth and yield in 3D suspension culturing^27–30,42–46^. Further, the hydrodynamic conditions are sensitive to many factors including the bioreactor design (e.g., impeller geometry, size and position, vessel geometry and size, positions of probes for pH, temperature and oxygen), medium viscosity and agitation rate^28,42^. They are currently not well understood and they are hard to control^28,42,45,46^. Moreover, how cells respond to the hydrodynamic conditions is not well known and is hard to study^28,45,46^. These knowledge gaps result in culture inconsistency and difficulty in scaling up cell production. We demonstrated that glioblastoma TICs expanded ~20 fold, yielding ~2 × 10^6^ cells/mL in dynamic 3D suspension culturing (Figs 7A,B and S7). With this yield, it will require about 5 liters of culturing, a culture volume that has not been demonstrated for TICs, to generate ~1 × 10^10^ glioblastoma TICs.

We propose that culturing conditions mimicking the *in vivo* 3D cell microenvironment that have no hydrodynamic stresses and uncontrolled cell agglomerations can significantly improve the cell culture efficiency. Accordingly, we designed the AlgTubes cell culture system for scalable culturing high quality and high quantity glioblastoma TICs. Unlike the traditional hydrogel-based cell culturing methods, in which cells are encapsulated and grown in hydrogel scaffolds^18,47,48^, in AlgTubes, cells are cultured in the free microspaces created by hydrogel tubes. The microspaces allow cells to efficiently interact with each other and expand, leading to high volumetric yield. The size of cell masses in AlgTubes is monodispersed and can be precisely controlled, which can significantly improve the culture homogeneity and efficiency. Cells in the AlgTubes are protected from the hydrodynamic stresses by the alginate shell, which can significantly reduce cell death.

The conceptual and technical innovations of AlgTubes lead to high culture efficiency. Primary glioblastoma TICs from different donors could be efficiently cultured in AlgTubes with high growth rate (~700-fold expansion/14 days) and high volumetric yield (~3.0 × 10^8^ cells/mL) (Figs 2 and S3). With this yield, it will take about 30 milliliters of AlgTubes to generate ~1 × 10^10^ glioblastoma TICs. After long term (~50 days, 10 passages) culture in AlgTubes, these cells still expressed the TIC markers Nestin, Sox2, CD44, Olig2, CD133 and CD15. The percentage of positive cells to these markers were not altered by the long-term culturing. It should be noted that using surface makers, such as CD133, to define glioblastoma TICs is still controversial^11,14–16^. For instance, the %CD133+ cells ranged from ~1% to ~96% and %CD15+ cells ranged from ~1% to ~48% in brain TICs from different patients^2^. Some other studies have questioned the utility of using CD133 as a marker for tumor-initiating cells^11,14–16,49–52^. Thus, we use three phenotype-based criteria to define glioblastoma TICs in this paper: (1) they can self-renew; (2) they can be differentiated into neural and glial cells; and (3) they can initiate tumors in *in vivo*. Our results showed TICs cultured for long-term in AlgTubes retained their capability to differentiate into neurons and glia cells (Figs 3, 4 and S4,5) or re-initiate tumors *in vivo* (Fig. 5). Additionally, AlgTubes-based bioreactors could be readily built for the scalable production of TICs (Fig. 6). Our comparative studies showed the AlgTubes offered significant advantages over the 2D, static 3D and dynamic 3D suspension culturing in terms of cell growth rate and yield (Fig. 7).

To our best knowledge, this is the first that utilizes alginate hydrogel tubes for successful TIC expansion. The AlgTubes will be of broad interest to individual laboratories, institutions and biotechnology companies working on developing new cancer therapies. AlgTubes can be a valuable tool for laboratories to maintain TICs. Due to its high volumetric yield and scalability, AlgTubes are particularly attractive for large-scale TIC production. A simple comparative calculation of producing ~1 × 10^12^ glioblastoma TICs from ~1 × 10^7^ seeds with 3D suspension culturing (e.g. the stirred-tank bioreactors) and AlgTubes shows the significant impact of AlgTubes’ high cell expansion per passage and volumetric yield (Fig. 7D). For the calculation, we assumed 20-fold expansion per 7 days per passage for stirred-tank bioreactors and 700-fold expansion per 14 days per passage for AlgTubes with the seeding density of 1 × 10^5^ cells/mL and 5 × 10^5^ cells/mL, respectively and a passaging efficiency (i.e., % of cells remaining viable after one passaging) of 80% for both. These assumptions are based on our research data. The production requires ~874 liters of total culture volume, 4 passaging operations and 28 days with stirred-tank bioreactors, which is technically and economically challenging (Fig. 7D). The production can be done with 4.49 liters of AlgTubes in 28 days and 2 passaging (Fig. 7D). The reductions in culture volume, culture time and passaging make the production technically feasible and also lead to enormous cuts in overall production cost.

The AlgTubes will also significantly advance the precision or personalized medicine. There are large variations between tumors or patients in terms of the genetics, epigenetics, cellular compositions and drug response or resistance^53^. The conventional method of using one treatment or drug (e.g. chemotherapy plus radiotherapy) to treat all patients has been proven to be of limited success. A better way is the precision or personalized medicine^54,55^. With this approach, tumor cells from each patient can be expanded *in vitro* and used to screen or test the existing drugs or their combination to find the best treatment for each patient. However, the widespread use of this approach highly relies on technologies that can efficiently and cost-effectively culture TICs for thousands of individuals. With AlgTubes, cells required for one patient (e.g. ~10^10^ cells) can be produced with about 30 mL alginate hydrogel tubes that can be contained in a closed 50 mL conical tube. Cells for many patients can be automatically produced with corresponding numbers of 50 mL tubes in parallel.

## Conclusion

In conclusion, we developed a new method for culturing glioblastoma TICs with high efficiency. It is simple, scalable and cost-effective. We believe the technology is a valuable tool for developing therapies targeted to TICs.

## Materials and Methods

### Materials

Cell culture reagents and their supplies: Neurocult^TM^ NS-A Proliferation kit (Stem cell technologies); Laminin (Invitrogen); Trypsin-EDTA (Invitrogen); Heparin and Trypsin inhibitor (Sigma); EGF and FGF (R&D). Syringe pump (New Era Pump System, Inc.); Disposable syringes (Henke sass wolf); Sodium Hyaluronate (Lifecore Biomedical); Sodium alginate (80~120 cp, Wako Chemicals); Calcium chloride (Acros Organics); Sodium Chloride (Fisher scientific). Mechanical stage and controller (CESCO); Bellows bottles (Spectrum Chemical Mfg. Corp.). Antibodies and their supplies: Tuj1 (1:10,000; Sigma); Nestin (1:200; Millipore); Ki-67 (1:500; Invitrogen); anti-glial fibrillary acidic protein (1:500; Dako); SOX2 (10 µg/mL; R&D system); Olig2 (20 µg/mL; Novus Biologicals); Calcein AM viability dye (eBiosicence); Ethidium homodimer I (Biotium); DAPI (Sigma). Trypan blue solution was obtained from Sigma-Aldrich. TRIzol (Ambion); Maxima first strand cDNA synthesis Kit (Thermo Fisher Scientific); Power SYBR Green Master Mix (Applied biosystems).

### Isolating primary TICs

All experiments were conducted in accordance with the national regulations. All experiments were approved by the Institutional Review Board (IRB) at University of Florida. All patients gave their informed consent before the experiments. Fresh brain tumor samples were acquired at the time of surgical excision. To establish these cell lines, the tumor samples were dissociated into single cells with 0.5% trypsin and then cultured in Neurocult^TM^ medium supplemented with bFGF, EGF and heparin^7^.

### 2D adherent culturing

For 2D adherent expansion, glioblastoma TICs were cultured in 6-well plates which were pre-coated with 10 μg/mL Laminin at 37 °C for 3 hours with Neurocult^TM^ medium supplemented with 10 ng/ml bFGF, 20 ng/ml EGF and 2 μg/mL heparin. The medium was changed daily and cells were passaged every 5 days. Briefly, cells were treated with 0.05% trypsin for 2–3 min at 37 °C and dissociated into single cells with a pipette. Then an equal volume of trypsin inhibitor was added to inactivate the trypsin. 1 × 10^5^ cells were usually passaged for each well of 6-well plate.

### 3D neurosphere culturing

Glioblastoma TICs neurospheres were treated with 0.05% trypsin at 37 °C for 5 min and dissociated into single cells with pipettes. Equal volume trypsin inhibitors were then added to inactivate the trypsin. 1 × 10^5^ cells/mL cells were seeded cells in non-treated T-25 Flask with Neurocult^TM^ medium supplemented with 10 ng/ml bFGF, 20 ng/ml EGF and 2 µg/ml heparin. The medium was changed daily and cells were passaged every 5 days.

### 3D suspension culturing in shaking plates

Glioblastoma TICs were suspended in low attached 6-well plate with Neurocult^TM^ medium supplemented with 10 ng/ml bFGF, 20 ng/ml EGF and 2 μg/mL heparin. The plates were shaken at 75 rpm. The medium was changed daily and cells were passaged every 5 days. For passage, TICs cells were collected via centrifuging at 200 g for 3 minutes and treated with 0.05% trypsin at 37 °C for 5 min before dissociating into single cells with pipettes. Equal volume trypsin inhibitors were then added to inactivate the trypsin.

### Processing AlgTubes

A micro-extruder was fabricated to process AlgTubes. 2% Hyaluronic acid (HA) solution containing single glioblastoma TICs is pumped into the central channel and 1.5% alginate solution in 145 mM NaCl is pumped into the side channel of the micro-extruder. The corresponding coaxial core-shell flow is extruded into a 100 mM Ca^2+^ buffer, which instantly crosslinks the alginates solution to form a hydrogel shell to make AlgTubes. Subsequently, cells laden AlgTubes were transferred to 6-well plate for culturing with the medium.

### Culturing glioblastoma TICs in AlgTubes

For a typical culture, 20 µL of glioblastoma TICs in the AlgTubes were suspended in 2 mL Neurocult^TM^ medium supplemented with 10 ng/ml bFGF, 20 ng/ml EGF and 2 µg/mL heparin in a 6-well plate and cultured at 37 °C with 5% CO_2_ and 21% O_2_. The medium was changed daily. To passage cells, the AlgTubes were dissolved with 0.5 mM EDTA for 5 minutes. The released cell mass was collected and treated with 0.05% trypsin at 37 °C for 5 minutes and dissociated into single cells with a pipette. Equal volume trypsin inhibitors were then added to inactivate the trypsin. Cell viability was qualitatively evaluated with live/dead cell staining according to the product manual (life technology). To quantify the viability, cells were stained with trypan blue and % of live cells were measured with a cell counter (TC20™, Bio-Rad).

### *In vitro* differentiation

For 2D differentiation, glioblastoma TICs cell masses were plated onto Laminin coated plates and cultured for two weeks in Neurocult^TM^ medium without bFGF, EGF or heparin. For 3D differentiation in the AlgTubes, glioblastoma TICs cell masses in AlgTubes were cultured for two weeks in Neurocult^TM^ medium without bFGF, EGF or heparin.

### Xenotransplantation

All animal protocols were approved by the Institutional Animal Care and Use Committee of the University of Nebraska-Lincoln. All experimental procedures involving animals were carried out in accordance with the guidelines of the Institutional Animal Care and Use Committee of the University of Nebraska-Lincoln. Glioblastoma TICs (~2 × 10^6^) were suspended in 25 µL PBS+ 25 µL Matrigel and injected subcutaneously at the back of the neck of the NOD-SCID mice. The tumors were harvested when sizes reached about 1.0 cm within 2 months. After fixed with 4% PFA for 48 hours, the tumors were cut into two halves. The first half was used for hematoxylin and eosin staining. Briefly, after sequentially dehydrated with 70%, 95% and 100% ethanol, the samples were embedded in paraffin and cut into 10 µm thick sections before staining. The second half was employed for immunostaining. Briefly, the samples were soaked in 20% sucrose for 7 days before embedded in OCT compounds and frozen. The samples were then cryosectioned for 10 µm thick sections before staining.

### Staining and imaging

To stain 2D surfaces glioblastoma TICs, cells were fixed with 4% paraformaldehyde (PFA) for 15 minutes and permeabilized with 0.25% Triton X-100 for 10 min and blocked with 5% goat serum for 1 hour. The samples were then incubated with primary antibodies at room temperature for 2 hours. After washed with PBS for 3 times, secondary antibodies in 2% BSA were added and incubated for another 1 hour before imaging. To stain 3D fibrous glioblastoma TICs in AlgTubes, cells were fixed with 4% PFA at room temperature for 1 hour, then incubated with PBS+ 0.25% Triton X-100+ 5% goat serum+ primary antibodies (Nestin, 1:200, Millipore; SOX2, 10 µg/mL, R&D system; Olig2, 20 µg/mL, Novus Biologicals; Tuj1, 1:10,000, Sigma; anti-glial fibrillary acidic protein, 1:500, Dako; Ki-67, 1:500, Invitrogen) at 4 °C for 2–3 days. After extensive washing, secondary antibodies (Alexa 488 Donkey anti-mouse, 1:500; Alexa 594 Donkey anti-rabbit, 1:500) in 2% BSA was added and incubated at 4 °C for 1 day. Cells were washed with PBS before imaging with NIKON A1 Confocal Microscopy. LIVE/DEAD^®^ cell viability staining was utilized to assess live and dead cells according to the product manual.

### Flow cytometry

We used the following antibodies: CD44-PE-Cy5 (1:100) (eBioscience), CD15-APC (1:5) (BD), CD133/2-PE (1:50) (Miltenyi Biotec). Annexin V-FITC Apoptosis Detection Kit was obtained from eBioscience. Staining was quantified by flow cytometry (Cytek DxP10).

### Quantitative real time polymerase chain reaction (qRT-PCR)

Total RNA was extracted from the harvested cells using TRIzol reagent. cDNA was synthesized using Maxima first strand cDNA synthesis kit according to the manufacturer’s instructions. Quantitative real time polymerase chain reaction was performed using Power SYBR Green Master Mix in an Eppendorf MasterCycler RealPlex4 (ThermoFisher Scientific). Experiments were performed in triplicate. The 2^−∆∆Ct^ method was employed to calculate the specific gene fold change of P10/P0, where ∆∆Ct = (Ct_target P10_-Ct_GAPDH P10_) - (Ct_target P0_-Ct_GAPDH P0_).

### Statistical analysis

The data are presented as the mean ± S.D. We employed an unpaired t-test to compare two groups and one-way ANOVA to compare more than two groups. P < 0.05 was considered statistically significant.

## Electronic supplementary material

Supplementary Information
