# Supplementary material for: Scalable Culturing of Primary Human Glioblastoma Tumor-Initiating Cells with a Cell-Friendly Culture System
Source: Sci Rep. 2018 Feb 23;8:3531. doi: 10.1038/s41598-018-21927-4 (PMC5824878; doi:10.1038/s41598-018-21927-4)
Supplement: Supplementary file 1 — Supplementary Information [file 41598_2018_21927_MOESM1_ESM.pdf]

Supplemental Information

Scalable Culturing of Primary Human Glioblastoma Tumor-Initiating Cells with a Cell-Friendly Culture System

Qiang Li[1,2], Haishuang Lin[1], Jack Rauch[1], Loic P. Deleyrolle[3], Brent A. Reynolds[3], Hendrik J. Viljoen[1], Chi Zhang[4], Chi Zhang[5], Linxia Gu[6], Erika Van Wyk[7] and Yuguo Lei[1,2,8,9]*

1: Department of Chemical and Biomolecular Engineering, University of Nebraska, Lincoln, Nebraska, USA
2: Biomedical Engineering Program, University of Nebraska, Lincoln, Nebraska, USA
3: Department of Neurosurgery, University of Florida College of Medicine, McKnight Brain Institute, Gainesville, Florida, USA
4: School of Biological Science, University of Nebraska, Lincoln, Nebraska, USA
5: Department of Radiation Oncology, College of Medicine, University of Nebraska Medical Center, Omaha, Nebraska, USA
6: Department of Mechanical & Materials Engineering, University of Nebraska, Lincoln, Nebraska, USA
7: CellGro LLC Adams, NE, USA
8: Mary and Dick Holland Regenerative Medicine Program, University of Nebraska Medical Center, Omaha, Nebraska, USA
9: Fred & Pamela Buffett Cancer Center, University of Nebraska Medical Center, Omaha, Nebraska, USA

* Corresponding Author
Yuguo Lei
820 N 16th St
Lincoln, NE 68588
Email : ylei14@unl.edu
Phone: 402-472-5313
Fax: 402-472-6989

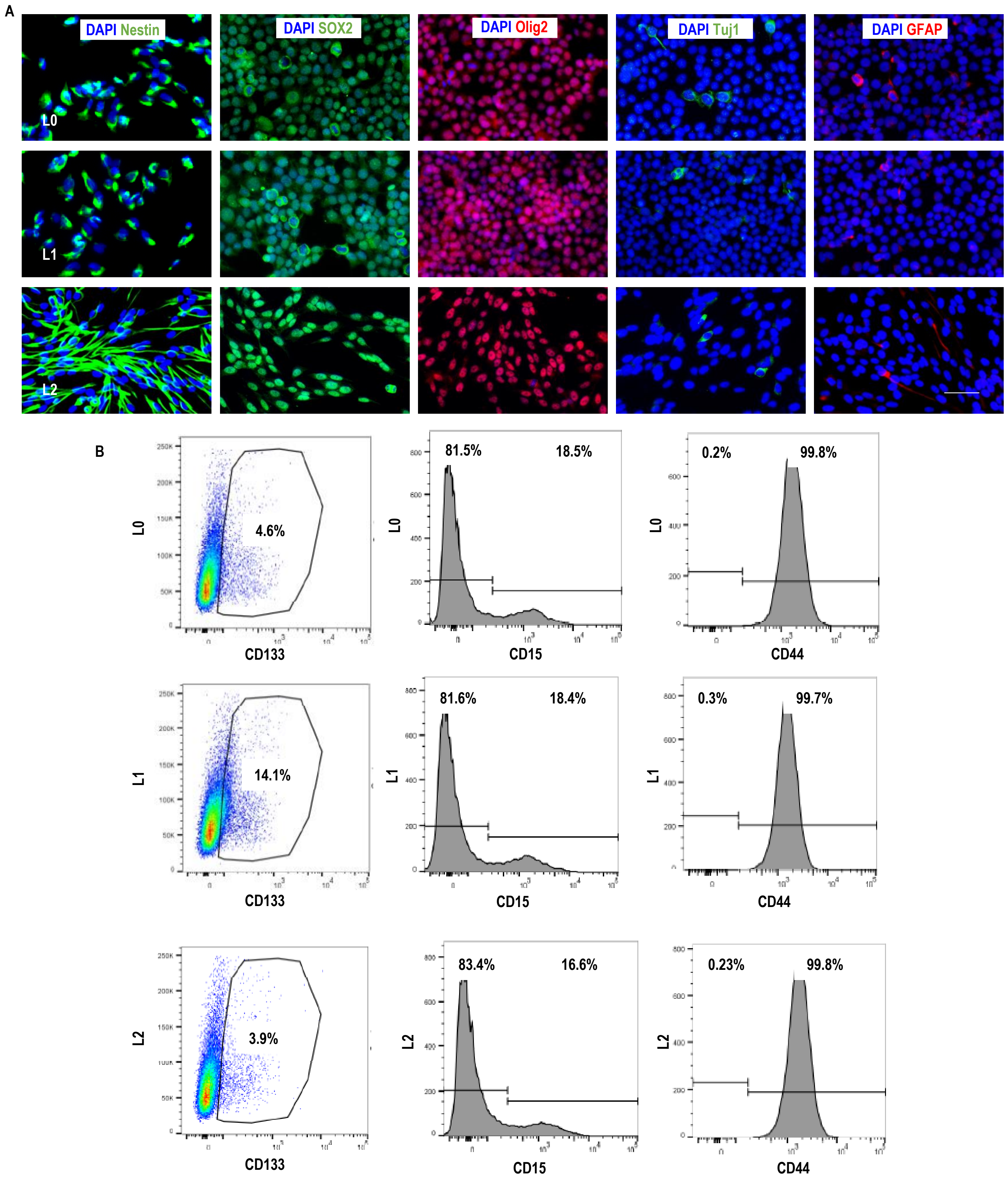

Figure S1. Characterization of the starting glioblastoma TICs (before cultured in AlgTubes). (A) Immunostaining of the TICs. Cells were plated on a Laminin-coated plate overnight before fixing and staining. Majority of TICs were Nestin+, SOX2+ and Olig2+, and few cells were Tuj1+ and GFAP+. Flow cytometry analysis for CD133, CD15 and CD44. Scale bar: 50 µm.

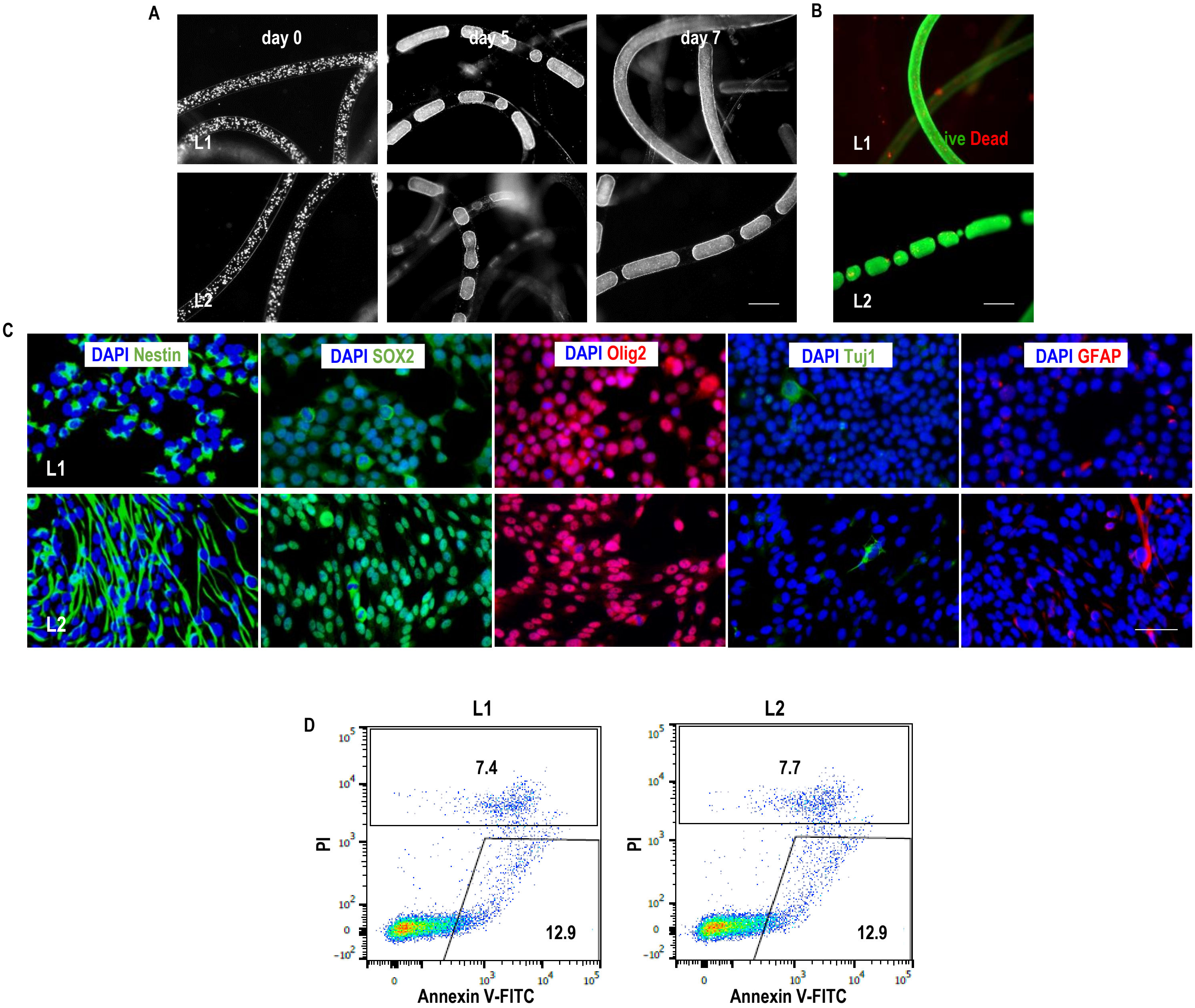

Figure S2. Culturing glioblastoma TICs in AlgTubes at passage 1 (L1 and L2 cells). (A) Microscopy images of glioblastoma TICs (L1, and L2) in AlgTubes on day 0, 5 and 7. (B) Live/dead staining of day 7 L1 and L2 cells in AlgTubes. (C) Immunostaining of the cultured TICs. Cells were released from AlgTubes on day 7 and plated on a Laminin-coated plate overnight before fixing and staining. Majority of L1 and L2 cells were Nestin+, SOX2+, and Olig2+. Few L1 and L2 cells were Tuj1+ and GFAP+. (D) Cell death was evaluated by Annexin V and PI staining after cultured in the AlgTubes for 7 days. Scale bar: (A, B) 400 µm, (C) 50 µm.

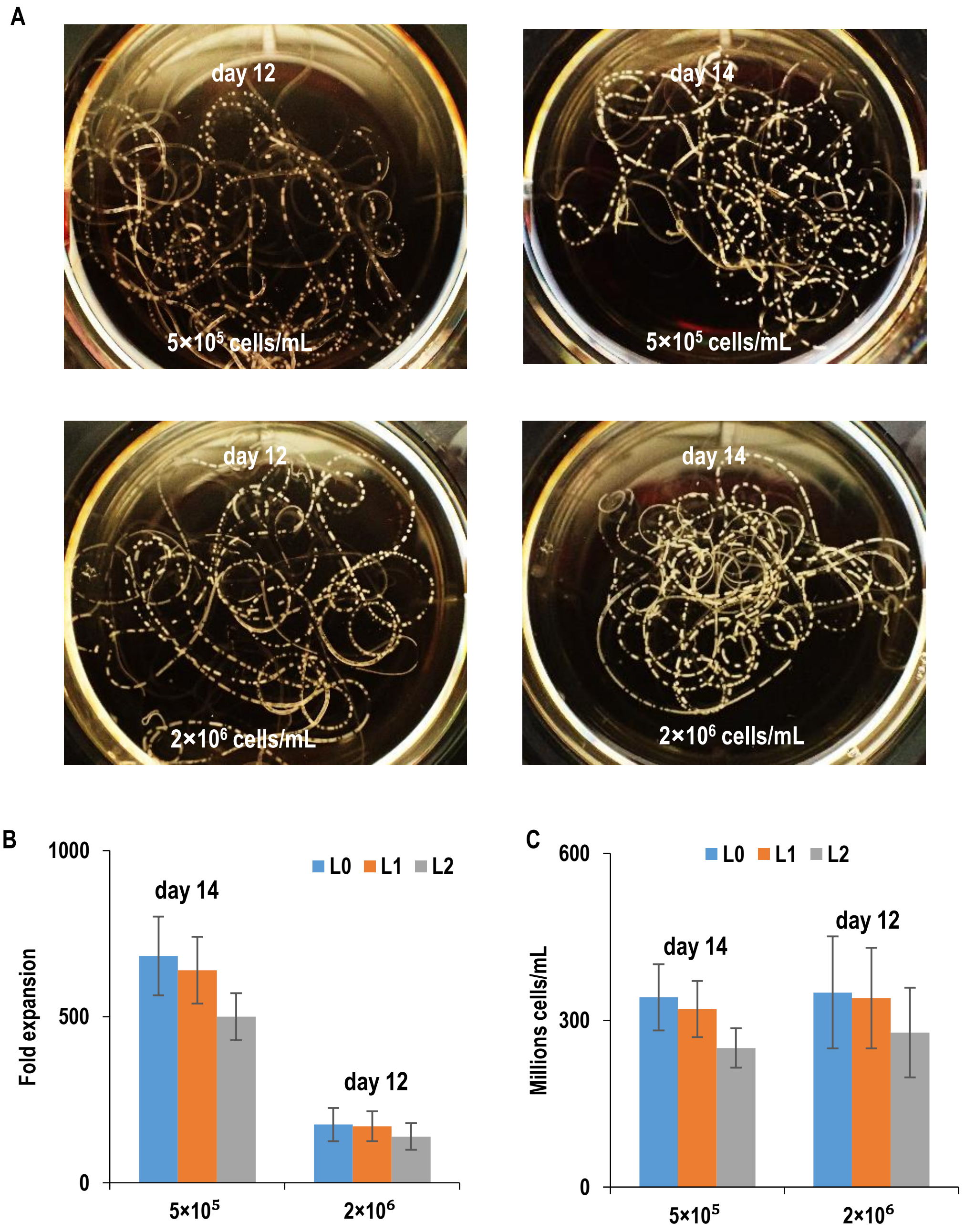

Figure S3. Culturing glioblastoma TICs in the AlgTubes. (A) Images of glioblastoma TICs (L0) on day 12 and day 14 when seeded at $5x10^5$ cells/mL and $2x10^6$ cells/mL. (B) The expansion fold of L0, L1 and L2 cells on day 12 when seeded at $2x10^6$ cells/mL, and day 14 when seeded at $5x10^5$ cells/mL. (C) The volumetric yield of L0, L1 and L2 cells on day 12 when seeded at $2x10^6$ cells/mL, and on day 14 when seeded at $5x10^5$ cells/mL. Error bars represent the standard deviation (n=3).

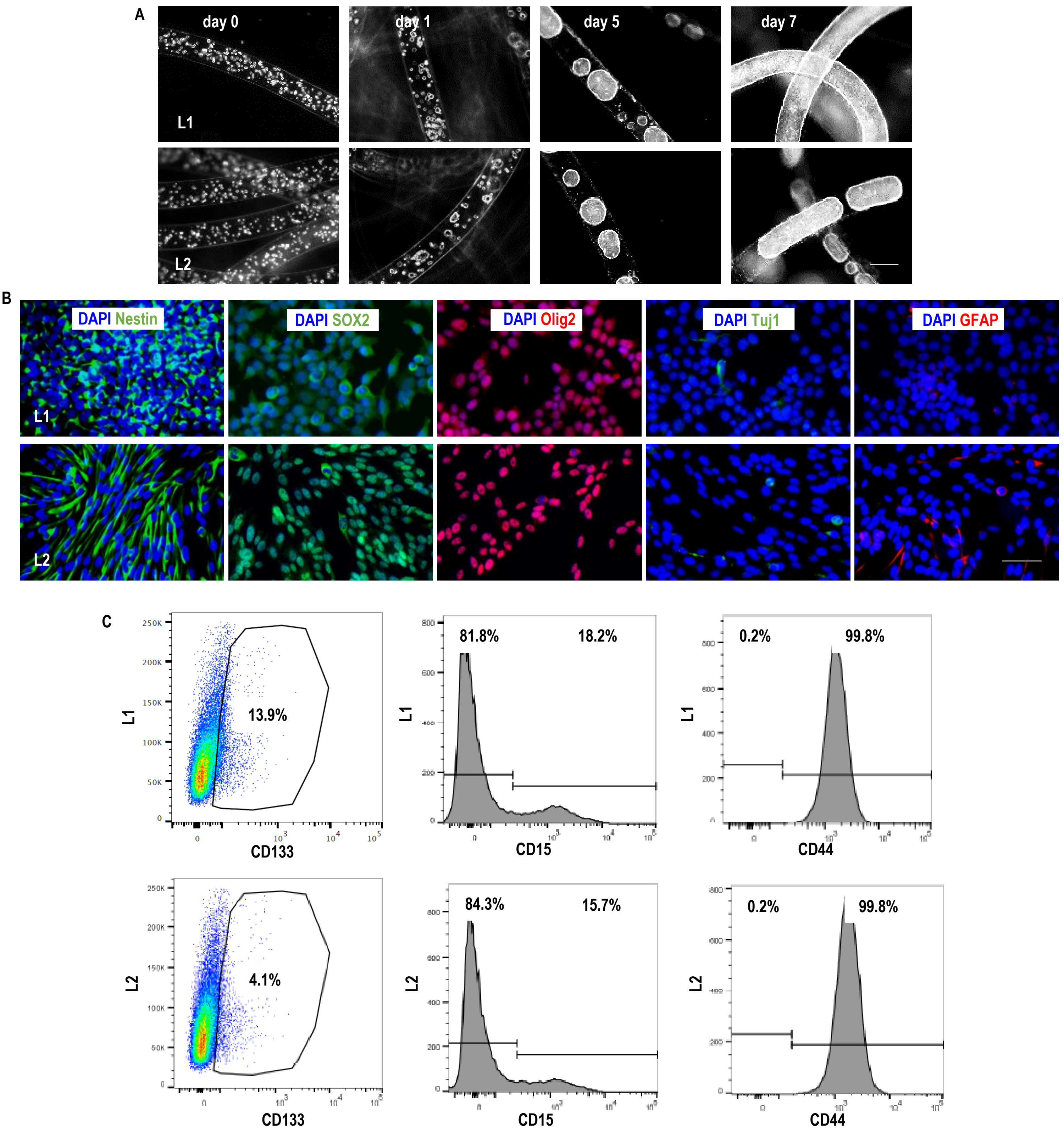

Figure S4. Long-term culturing of glioblastoma TICs in AlgTubes (passage 10).

D

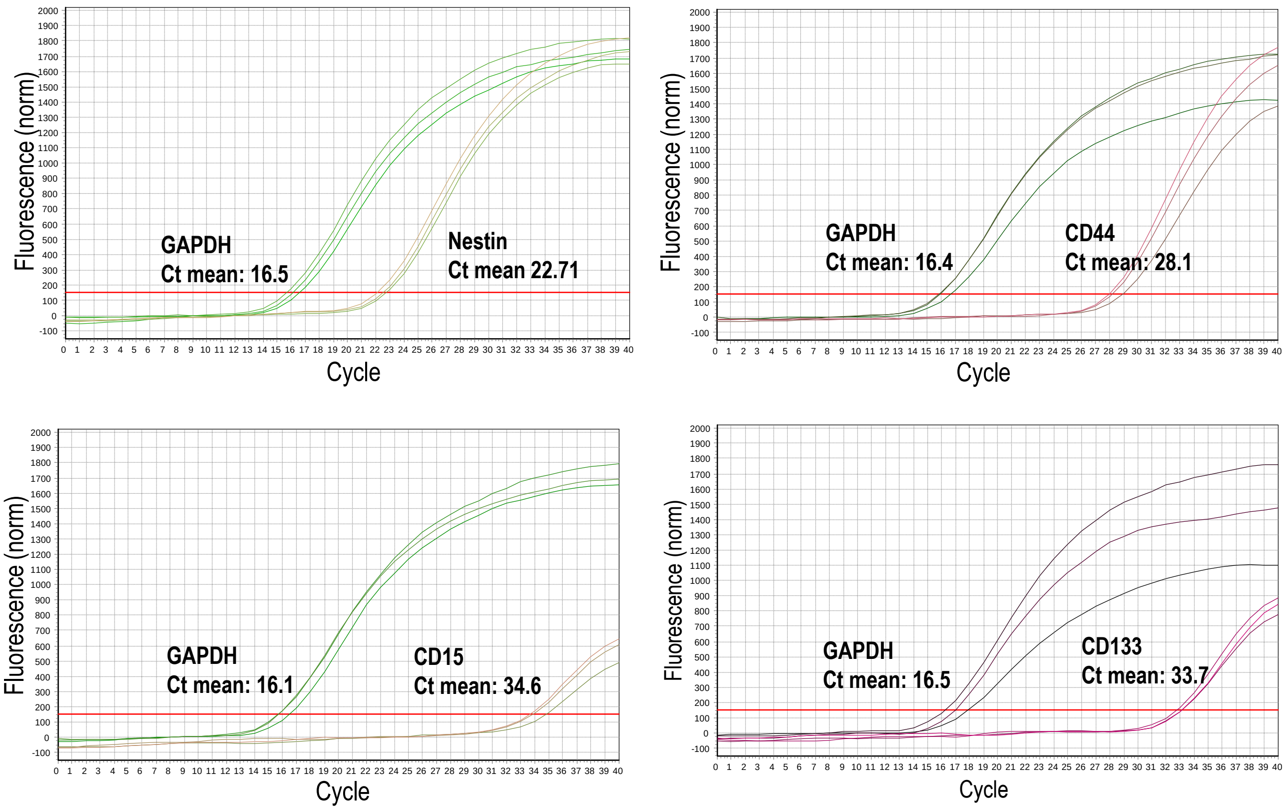

E

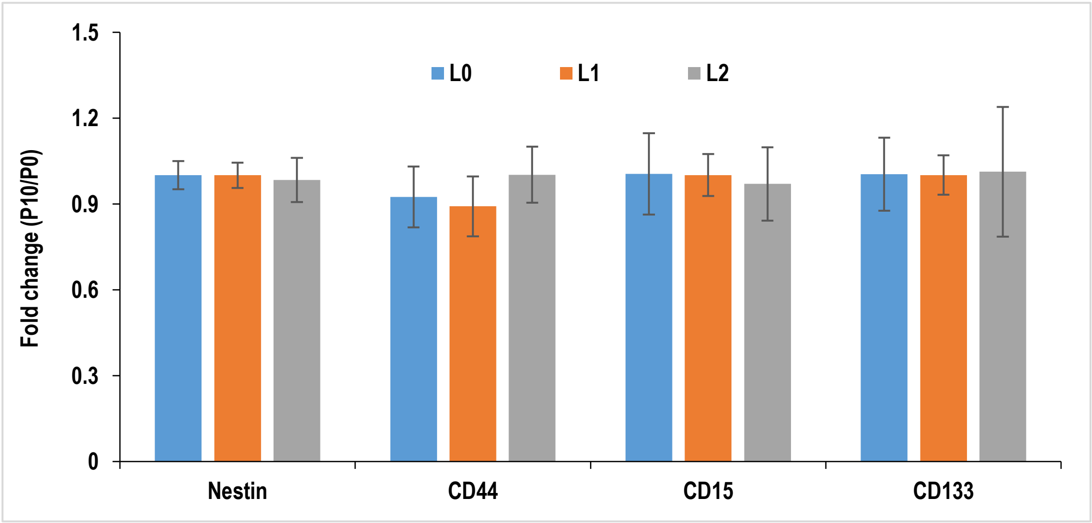

Figure S4. Long-term culturing of glioblastoma TICs in AlgTubes (passage 10). (A) Microscopy images of L1, and L2 on day 0, 1, 5 and 7 at passage 10 (B) Immunostaining of the cultured TICs. Cells were released from AlgTubes on day 7 at passage 10 and plated on a Laminin-coated plate overnight before fixing and staining. Majority of L1 and L2 cells were Nestin+, SOX2+, and Olig2+. Few L1 and L2 cells were Tuj1+ and GFAP+. (C) Flow cytometry analysis showed that 13.9% L1 cells were CD133+, 18.2% L1 cells were CD15+ and 99.8% L1 cells were CD44+ ; 4.1% L2 cells were CD133+, 15.7% L2 cells were CD15+ and 99.8% L2 cells were CD44+. (D) qRT-PCR amplification curves of GAPDH, Nestin, CD44, CD15, and CD133 of passage 10 L0 cells cultured in AlgTubes. (E) qRT-PCR on the mRNA level of Nestin, CD44, CD15, and CD133. The ratio of their expression at passage 10 and passage 0 were shown. Error bars represent the standard deviaton (n=3). Scale bar: (A) 200 µm; (B) 50 µm.

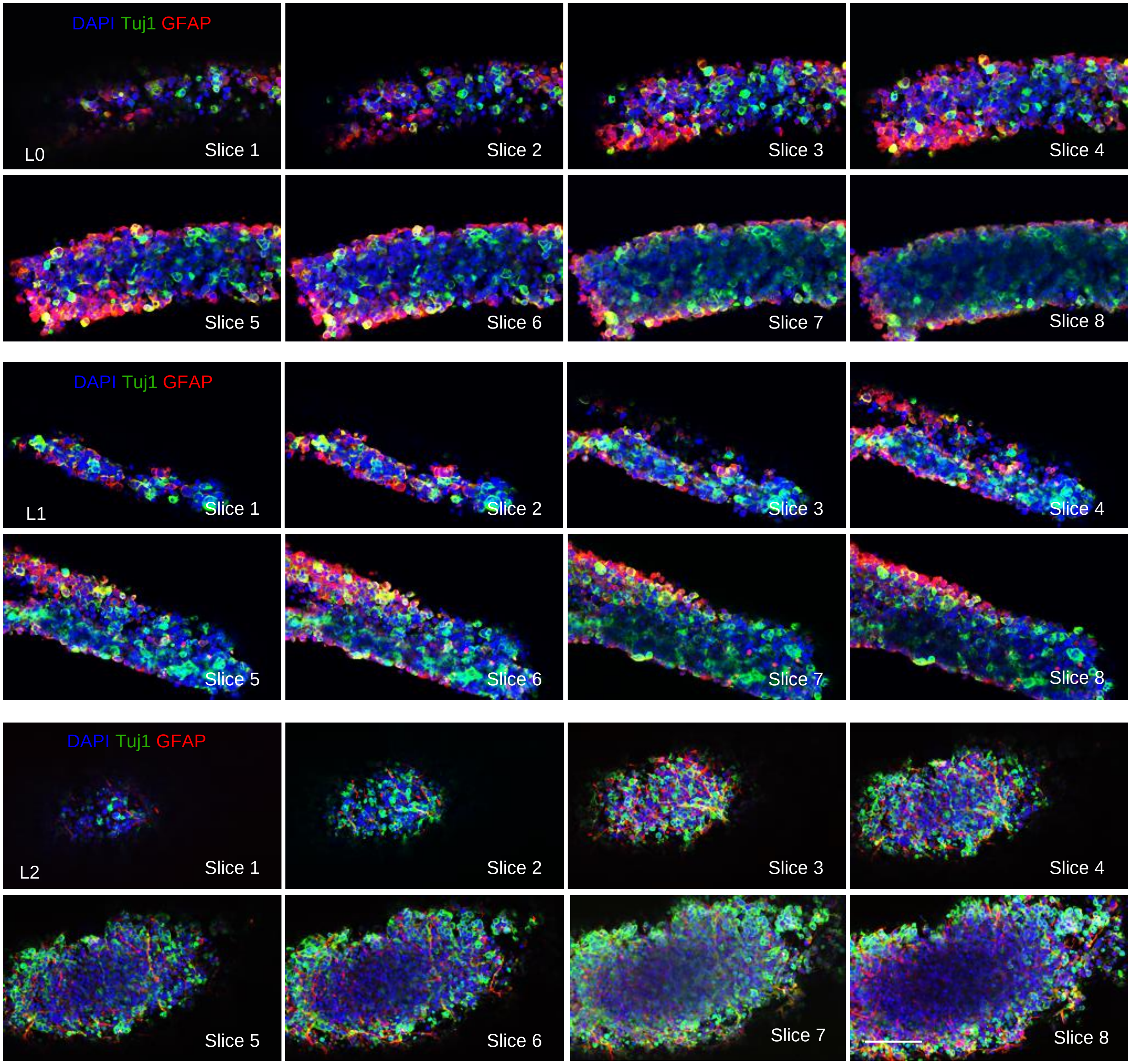

Figure S5. *In vitro* differentiation of glioblastoma TICs. After culturing 10 passages in AlgTubes, glioblastoma TICs were differentiated for 14 days in AlgTubes and stained with antibodies against neuron marker, Tuj1 and glia marker, GFAP. Confocal images of varied slices of a fibrous cell mass were shown. Scale bar: 100 µm.

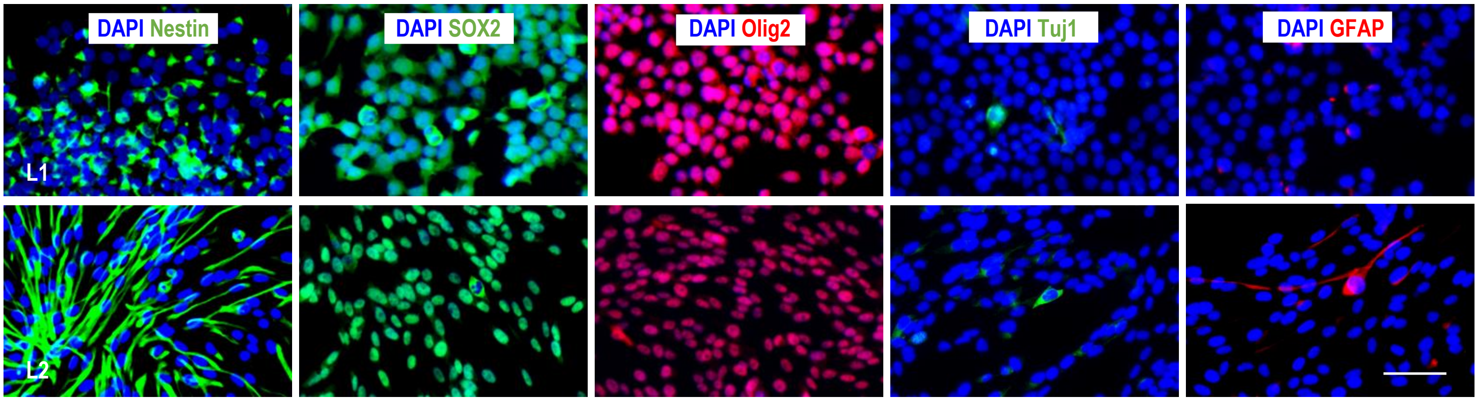

Figure S6. A prototype bioreactor for scalable TICs production (L1 and L2 cells). Glioblastoma TICs harvested from the bioreactor on day 10. Immunostaining of the cultured TICs. Majority of L1 and L2 cells were Nestin+, SOX2+, and Olig2+. Few L1 and L2 cells were Tuj1+ and GFAP+. Scale bar: 50 µm.

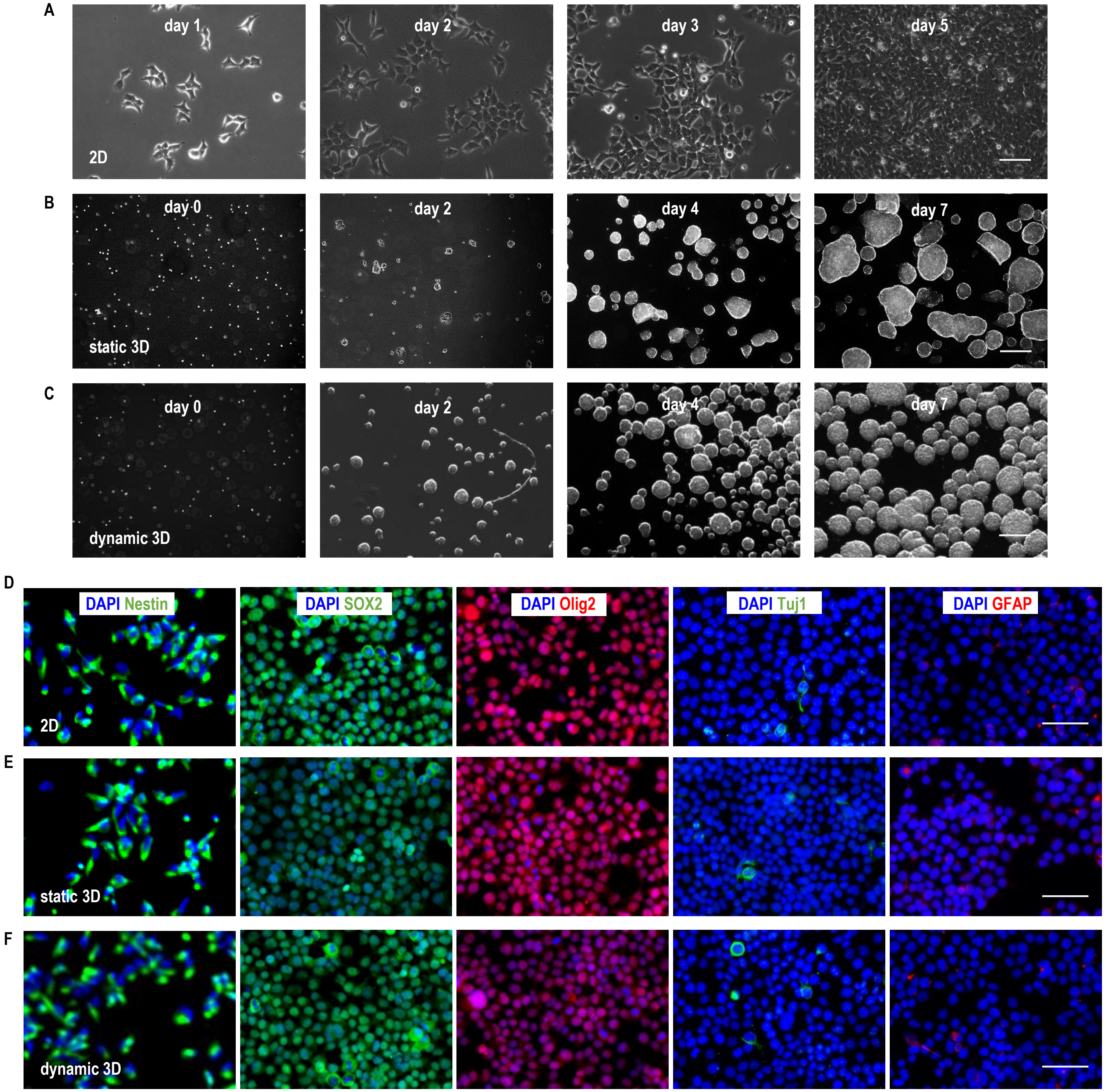

Figure S7. Comparing culturing glioblastoma TICs in AlgTubes, 2D, static and dynamic 3D suspension culturing. (A) Phase images of glioblastoma TICs (L0) in 2D culturing. Scale bar: 50 µm. (B) Phase images of glioblastoma TICs (L0) in static 3D suspension culturing. Scale bar: 250 µm. (C) Phase images of glioblastoma TICs (L0) in dynamic 3D suspension culturing. Scale bar: 250 µm. (D-F) Immunostaining of the cultured TICs (L0). Cells from 2D, static 3D, and dynamic 3D were plated on a Laminin-coated plate overnight before fixing and staining. Majority of cells were Nestin+, SOX2+, and Olig2+. Few cells were Tuj1+ and GFAP+. Scale bar: 50 µm.
